# Metabolic changes with the occurrence of atherosclerotic plaques and the effects of statins

**DOI:** 10.3389/fimmu.2023.1301051

**Published:** 2023-12-08

**Authors:** Lingmin Zhao, Di Ma, LiJuan Wang, XingQi Su, LiangShu Feng, LiChong Zhu, Ying Chen, YuLei Hao, XinYu Wang, JiaChun Feng

**Affiliations:** Bethune First Hospital, Jilin University, Changchun, China

**Keywords:** atherosclerosis plaque, cell metabolism, inflammatory response, statins, anti-inflammatory drug, cardiovascular and cerebrovascular diseases

## Abstract

Atherosclerosis is a common cardiovascular disease caused by the abnormal expression of multiple factors and genes influenced by both environmental and genetic factors. The primary manifestation of atherosclerosis is plaque formation, which occurs when inflammatory cells consume excess lipids, affecting their retention and modification within the arterial intima. This triggers endothelial cell (EC) activation, immune cell infiltration, vascular smooth muscle cell (VSMC) proliferation and migration, foam cell formation, lipid streaks, and fibrous plaque development. These processes can lead to vascular wall sclerosis, lumen stenosis, and thrombosis. Immune cells, ECs, and VSMCs in atherosclerotic plaques undergo significant metabolic changes and inflammatory responses. The interaction of cytokines and chemokines secreted by these cells leads to the onset, progression, and regression of atherosclerosis. The regulation of cell- or cytokine-based immune responses is a novel therapeutic approach for atherosclerosis. Statins are currently the primary pharmacological agents utilised for managing unstable plaques owing to their ability to enhance endothelial function, regulate VSMC proliferation and apoptosis by reducing cholesterol levels, and mitigate the expression and activity of inflammatory cytokines. In this review, we provide an overview of the metabolic changes associated with atherosclerosis, describe the effects of inflammatory responses on atherosclerotic plaques, and discuss the mechanisms through which statins contribute to plaque stabilisation. Additionally, we examine the role of statins in combination with other drugs in the management of atherosclerosis.

## Graphical Abstract

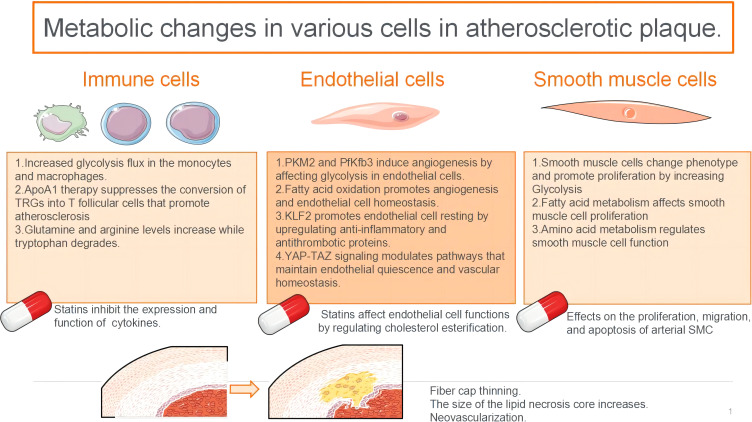

## 1 Introduction

Atherosclerosis is a common cardiovascular disease. It primarily manifests in the intima of affected arteries as lipid depositions, infiltrations of monocytes and lymphocytes, the migration and proliferation of vascular smooth muscle cells (VSMCs), and the formation of foam cells, lipid striations, and fibrous plaques, which further contributes to vascular wall sclerosis, stenosis, and thrombosis. Atherosclerosis can be asymptomatic for decades; however, when complications occur, such as the rupture of an atherosclerotic plaque, it can lead to myocardial infarction, stroke, peripheral vascular disease, and other high-fatality conditions. Treatment of these disease complications often relies on pharmaceutical interventions. In this review, we summarise the metabolic changes in some of the major cell types involved in atherosclerotic plaques and discuss the mechanisms, side effects, and progression of atherosclerotic plaques when treated with statins.

## 2 Metabolic changes with the occurrence of atherosclerosis

During the development of atherosclerotic lesions, the enhanced permeability of endothelial cells (ECs)facilitates the infiltration of peripheral inflammatory cells into the plaque (1). Macrophages, T cells, dendritic cells(DCs), and B cells are the most common types of immune cells found in growing arteriosclerotic plaques (2). Data from animal models show that selectively depleting or modulating the function of immune cells involved in atherosclerosis, inhibiting or blocking specific cytokines involved in inflammation and plaque development and regulating the immune cell bank and the secreted mediators in the arterial wall can impact atherosclerosis (2). Clinical trials, such as CANTOS (Canakinumab Anti-inflammatory thrombosis results study) and LoDoCo2 (low-dose colchicine for secondary prevention of stable coronary artery disease patients), also strongly suggest that immune regulation may be a relevant treatment option for human atherosclerosis (3, 4).

Metabolic regulation is closely related to the induction of immune responses. Metabolism in the microenvironment affects the proliferation and differentiation of immune cells and promotes the synthesis and secretion of immune mediators. In this section, we summarise the process of metabolic reprogramming in immune cells, particularly focusing on energy-related metabolic pathways. Moreover, we explore the potential of targeting the immune metabolism of macrophages and lymphocytes to control inflammatory responses in atherosclerotic lesions. The hypoxic environment within atherosclerotic plaques initially stimulates macrophage polarisation and enhances macrophage glycolysis.

### 2.1 Metabolic changes in macrophages, T and B cells that affect inflammation

#### 2.1.1 Increased glycolysis flux in the monocytes and macrophages of patients with atherosclerosis

**Figure 1:**
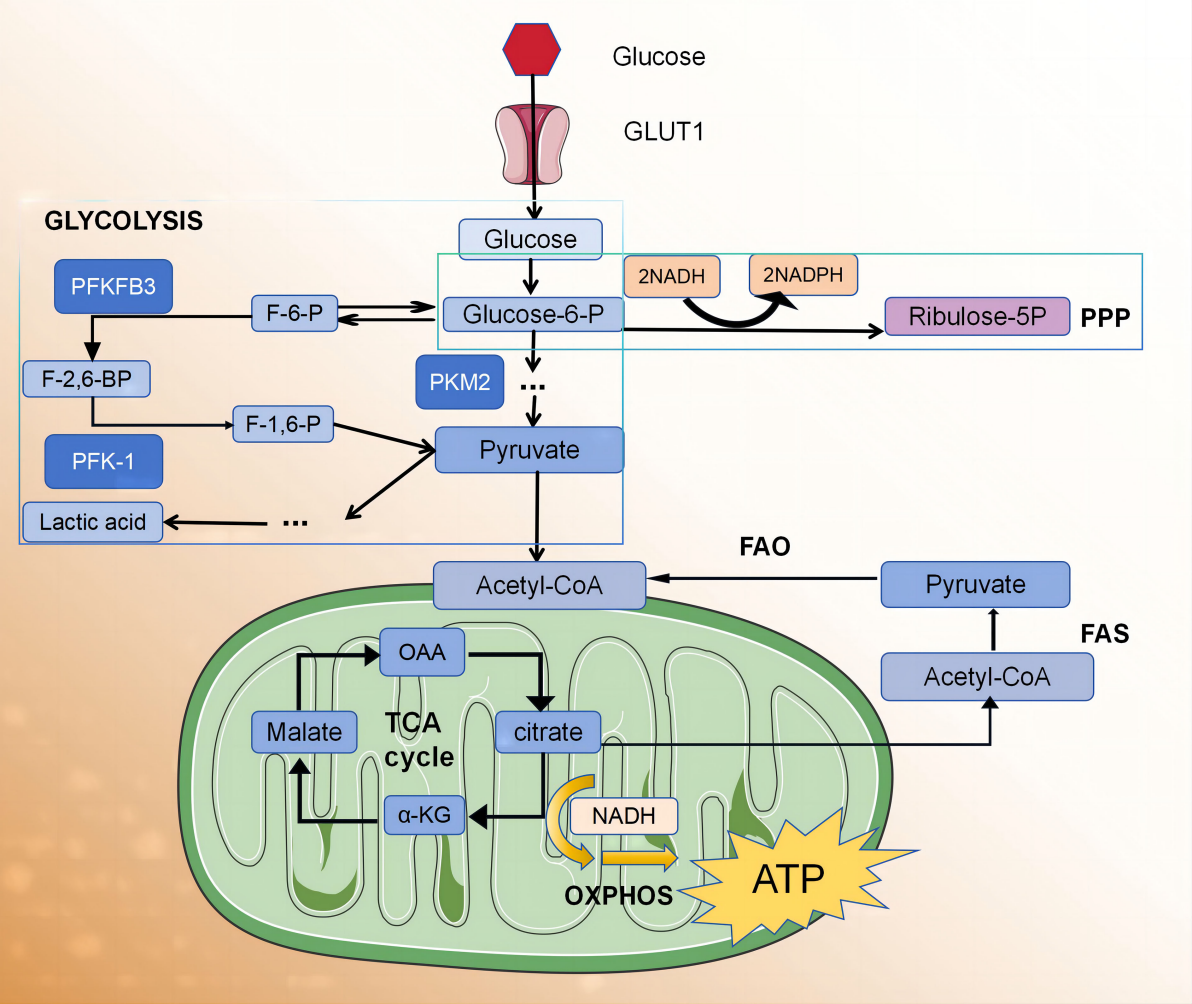
The glycolytic fluxes of immune cells, endothelial cells and smooth muscle cells in atherosclerotic plaque were increased.

Adenosine triphosphate (ATP) is the universal energy currency within cells. The main sources of ATP for macrophages and lymphocytes involve glucose metabolism via glycolysis and the pentose phosphate pathway (PPP). Under normal oxygen conditions, cells generate 36 ATP molecules through the citric acid cycle and oxidative phosphorylation (OXPHOS) pathway in the mitochondria. In an anaerobic environment, pyruvate is reduced as a hydrogen acceptor to lactate, resulting in a decrease in ATP production (two molecules) but an increase in ATP production rate. In the 1920s, the German scientist Warburg observed that, even in the presence of sufficient oxygen, tumour cells suppress aerobic respiration through a series of molecular mechanisms and promote efficient glycolysis reactions. This metabolic shift leads to the production of a large amount of ATP, creating a microenvironment suitable for the survival of tumour cells. This unique form of energy metabolism is known as the “Warburg effect”. In recent years, several research groups have observed that the metabolic profile of activated macrophages, induced by phagocytosis or inflammatory stimulation, undergoes reprogramming. This reprogramming involves a transition from oxidative metabolism to “Warburg metabolism” (5), enabling the rapid supply of energy required for the inflammatory process and the essential metabolic intermediates needed for biosynthesis (**Figure 1**).

##### 2.1.1.1 Macrophage metabolism

Nonpolarized macrophages, under steady-state conditions, primarily obtain energy via the OXPHOS pathway. However, the metabolic characteristics of polarised macrophages (M1 and M2) when obtaining energy are relatively complex (6), and they exhibit distinct metabolic profiles. According to the stimulation received from the microenvironment, M1 macrophages use glycolysis and the PPP to meet their ATP requirements. Simultaneously, OXPHOS and fatty acid oxidation (FAO) in the citric acid cycle are downregulated (7–10). In contrast, M2 macrophages exhibit a metabolic profile characterised by a complete citric acid cycle and enhanced FAO and OXPHOS (11, 12).

Glycolysis serves as a fundamental mechanism for energy production within cells. The glycolytic pathway occurring in the cytoplasm facilitates the conversion of glucose into pyruvate, yielding two ATP molecules per glucose unit. It also provides metabolic intermediates required for biosynthetic pathways, including ribose, amino acid, and fatty acid synthesis. The PPP operates in conjunction with the glycolysis pathway, harnessing the energy derived from glucose 6-phosphate conversion to ribulose 5-phosphate for NADP+ reduction to nicotinamide adenine dinucleotide phosphate (NADPH). This enzymatic process generates reactive oxygen species (ROS), which exhibit pathogenic activity. Additionally, high levels of NADPH are essential for maintaining the reduced form of the antioxidant glutathione, which protects cells from oxidative stress.

The adaptation of glycolysis metabolism depends on the activation of several transcription factors, including hypoxia-inducible factor 1 (HIF1α) (13). HIF1α regulates the expression of glycolytic enzymes, glucose transporter 1 (GLUT1), and genes encoding inflammatory mediators (13–15). The upregulation of GLUT1 promotes rapid glucose uptake by M1 macrophages (10). Moreover, HIF1α supports the conversion of pyruvic acid to lactic acid by promoting the expression of lactate dehydrogenase (LDH) (16) and pyruvic acid dehydrogenase kinase (PDK) (17, 18), which are critical for restoring NAD+ levels and maintaining glycolytic flux. HIF1α regulation in macrophages predominantly occurs through two main signalling pathways, involving α expression of several genes, including toll-like receptor (TLR)/nuclear factor-κB (NF-κB) (15) and AKT/mammalian target of rapamycin (mTOR) (17–19) pathways. AKT kinase regulates macrophage polarisation in a subtype-specific manner, with AKT1 deletion promoting the M1 spectrum, while AKT2 deletion amplifies the M2 reaction (17). The other two factors that regulate glycolytic flux in M1 macrophages are 6-phosphofructose-2-kinase/fructose 2,6-diphosphatase 3 (Pfkfb3) and pyruvate kinase M2 (PKM2). Pfkfb3 catalyses the conversion of fructose-2,6-diphosphate to fructose-6-phosphate with low efficiency, thereby enhancing glycolytic flux. Moreover, M1 cells upregulate subtype PKM2, which, when overexpressed, exists in a balance between enzyme-inactive monomers or dimers and enzyme-active tetramers (20). The inactive enzymes translocate into the nucleus and bind to HIF1α, triggering the expression of HIF1α-regulated genes, whereas the enzymatically active tetramers promote glycolysis and M1 polarisation in the cytoplasm (20). In M1 macrophages, glycolysis significantly impacts macrophage functions, including phagocytosis, ROS production, and proinflammatory cytokine secretion (21). However, the role of glycolysis in M2 macrophages remains a subject of debate. Several studies have proposed that the presence of 2-deoxyglucose, an inhibitor of glycolysis, may impede M2 polarisation and impair its functionality (22, 23). There are also data indicating that M2 differentiation relies more on OXPHOS than glycolysis (24, 25). The tricarboxylic acid cycle (TCA cycle) is also complete in M2 macrophages.

Early studies using 18 fluorodeoxyglucose (18 FDG) positron emission tomography (PET) revealed elevated glucose uptake in atherosclerosis plaques of rabbits and humans compared to healthy blood vessels. Additionally, macrophage glycolysis levels, PPP activity, and TCA cycle metabolites were increased within plaques (12, 26). Notably, *in vitro* studies demonstrated that exposure to inflammatory factors, rather than a hypoxic environment, leads to a reduction in TCA cycle metabolism in polarized M1 macrophages, while the glycolytic metabolism level does not concurrently increase. These findings support the hypothesis that hypoxic stimulation enhances glycolysis in M1-polarized macrophages within atherosclerotic arteries (27). However, whether nonhypoxic stimulation enhances glycolysis in macrophages remains controversial (28).

##### 2.1.1.2 T cell metabolism

Several studies have indicated that natural T cells are activated and differentiated into different subgroups after entering atherosclerotic plaques, and different T cell subgroups play distinct roles (29). For instance, a study has shown that the absence of CD8^+^ T cells in mice did not affect plaque size, but immunotherapy with ApoB100 P210 peptide in CD8^+^ *ApoE^-/-^* mice mitigated atherosclerotic lesions, suggesting that CD8^+^ T cells may have a different function (30). The role of CD4^+^ T cells in atherosclerosis is multifaceted. Static CD4^+^ T cells differentiate into effector T cells (Teff cell) and regulatory T cells (Treg cell) upon activation. Different Teff cell lines proliferate and differentiate rapidly depending on their microenvironment (31).

Effector CD4^+^ T cells in plaques primarily include Th1, Th2, and Th17 cells. Factors involved in the Th1 reaction, including tumour necrosis factor-α (TNF-α), recombinant human interferon-γ (IFN-γ), interleukin (IL)-12, and IL-18, have been proven to promote atherosclerosis through leukocyte recruitment, EC injury, and oxidative stress (32–36). However, the role of Th17 cells in atherosclerosis remains controversial. A study analysing atherosclerotic plaques in human coronary arteries showed that the cytokine IL-17 released by Th17 can synergistically increase the secretion of IL-6 with IFN-γ, promoting inflammation and atherosclerosis (37, 38). Conversely, in a study by Madhur MS et al., IL-17A did not affect atherosclerotic plaque burden in IL17A*ApoE^-/-^* mice fed a high-fat diet (39, 40). IL-10 secreted by Th17 cells may prevent the recruitment of T cells and macrophages and enhance protection against atherosclerosis by promoting the transformation of macrophages from an inflammatory to an anti-inflammatory phenotype (41, 42). The role of Th2 cells in the development of atherosclerosis remains uncertain. Th2 cells secrete IL-5, IL-10, and IL-13 and activate B cells to produce antibodies, which are believed to counteract the pro-atherosclerosis effects of Th1 cells. Furthermore, a study has shown that IL-4, an autocrine growth factor of Th2 cells, did not significantly affect the development of atherosclerotic lesions in *ApoE^-/-^* or female *ldlr^-/-^* mice (43). However, another study found that IL-4 deficiency reduced the formation of atherosclerotic lesions in female *ldlr^-/-^* mice (44). Therefore, the role of IL-4 in atherosclerotic lesions remains to be further clarified. Treg cells increase plaque stability mainly by secreting transforming growth factor-β (TGF-β) and IL-10. TGF-β can inhibit the recruitment and activation of T cells and macrophages, promote the proliferation of VSMCs, and maintain atherosclerotic lesion stability (31, 45). The balance between Teff and Treg cell subsets and their respective functions significantly influences the development of atherosclerotic lesions (46). Therefore, maintaining the equilibrium of the Treg/Teff ratio and function is essential to prevent the onset of atherosclerosis and slow its progression.

As T cells transition from a quiescent state to an activated state within atherosclerotic lesions, glycolysis becomes imperative for rapid energy generation. While OXPHOS is the primary pathway for energy generation in static T cells (31, 45), activated T cells require the GLUT1 receptor to increase glucose uptake and promote the upregulation of glycolytic enzymes to catalyse glycolysis, resulting in increased pyruvic acid production (47). Subsequently, pyruvic acid can be converted into lactic acid within the cytoplasm through LDH or into acetyl coenzyme A within the mitochondria through pyruvate dehydrogenase (PDH). Additionally, the NADH and flavin adenine dinucleotide (FAD) generated during this process are primarily produced within the mitochondria and ATP is produced through OXPHOS.

Teff cells (Th1, Th2, and Th17) predominantly rely on glycolytic metabolism and glutamine catabolism for energy, whereas Treg cells primarily depend on FAO (48). This metabolic distinction between Teff and Treg subpopulations plays a pivotal role in governing the differentiation fate of CD4^+^ T cells and maintaining optimal immune function (46, 49). Michalek et al. (48) differentiated Th1, Th2, and Th17 cells *in vitro* and found increased glycolysis and GLUT1 expression in these cells. Compared to wild-type mice, GLUT1 transgenic mice showed increased glucose uptake and selective enrichment of Teff cells. Conversely, inhibiting glycolytic metabolism through treatments like 2-deoxyglucose, a hexokinase inhibitor, reduces Teff cell production and impairs immune function *in vitro* and *in vivo* (13, 48, 50, 51). These findings indicate that the Teff subpopulation undergoes increased glycolytic metabolism, a necessity for differentiation and functional specialisation.

The high activity level of PDK1 in Th17 cells leads to higher extracellular acidification and glycolysis rates than in Th1 cells (46). Furthermore, PKM2 serves as the ultimate rate-limiting enzyme in CD4^+^ T cell glycolysis. Hyperhomocysteinemia expedites the onset and progression of atherosclerosis by augmenting both PKM2 protein expression and activity in *ApoE^−/−^* mice (52). Therefore, targeted inhibition of the PKM2 metabolic pathway in CD4^+^ T cells may represent a novel strategy for treating atherosclerotic lesions.

Glycolysis promotes the activation, proliferation, and migration of Treg cells to inflammatory tissues (53–55). The expression of the Treg transcription factor, forkhead box protein 3 (FOXP3), suppresses glycolytic metabolism and enhances OXPHOS by downregulating Myc protein expression. This metabolic adaptation allows Treg cells to function effectively in a low-glucose/high-lactate environment (56). However, the anoxic conditions within plaques may decrease FOXP3 expression and impair the protective effects of Treg cells on atherosclerosis (57, 58). In summary, the induction of the glycolytic pathway plays a crucial role in modulating the differentiation balance between Treg and Teff cells. However, further research is required to elucidate how the atherosclerotic plaque microenvironment affects the metabolic and functional properties of distinct T-cell subsets (**Table 1**).

**Table 1:** T cell typing and function.

T cell type	The main secretory factor	Effects in lesions		Contradiction
CD8^+^ T cell	TNF-α, IFN-γ	Cytotoxic function	Plaque promotion (30)	
	IL-5, IL-10,IL-13	Modulating immune effects and assisting immune responses	Inhibition of plaque lesions (30)	
Th1	TNF-α, IFN-γ, IL-12, IL-18	Affecting leukocyte recruitment, EC damage and oxidative stress	Plaque promotion (33–36)	
Th2	IL-5, IL-10,IL-13	Activating B cells to produce antibodies	Inhibition of plaque lesions	
	IL-4	Did not affect the development of atherosclerotic lesions (43)		Plaque promotion (44)
Th17	IL-17	synergistically increase the secretion of IL-6 with IFN-γ	Plaque promotion (37, 38)	Did not affect the development of atherosclerotic lesions (40, 41)
Treg	TGF-β,IL-10	Inhibiting the recruitment and activation of T cells and macrophages, promote the proliferation of VSMCs	Inhibition of plaque lesions (31, 45)	

TNF, tumor necrosis factor; IFN, Human Interferon; IL, Interleukin; TGF, transforming growth factor; Th, helper T; Treg, regulatory T.

##### 2.1.1.3 DCs and B cells

In atherosclerotic lesions, vascular DCs are present within the plaque and outer membrane (59). Apart from their role in lipid absorption and clearance of apoptotic cells in plaques, DCs also facilitate T cell activation and proliferation by presenting antigens derived from autologous sources. DCs secrete a diverse array of cytokines that indirectly modulate the functionality of other immune cells, thereby contributing to immune-mediated vascular wall damage and the development of atherosclerosis, including their impact on B-cell activation (60).

B cells play a significant role in the development of atherosclerosis (61); however, there is ongoing debate regarding their specific involvement in plaque formation (62). B cells can be categorised into two subtypes: B1 and B2. The former produces natural antibodies (IgM) that are believed to exert protective effects against atherosclerosis by inhibiting necrotic nucleus formation on blood vessel walls (63, 64). Conversely, B2 cells participate in adaptive immune responses and secrete cytokines IL-10 and TNF-α, which influence Treg development and potentially promote the formation of atherosclerosis (65). Studies have investigated approaches such as anti-CD20 depletion or the absence of the BAFFR receptor to mitigate damage caused by B2 cell activation and protect hypercholesterolemic mice from developing atherosclerosis (66–68).

Glycolysis plays a crucial role in the activation of B cells within atherosclerotic lesions. The B-cell receptor (BCR), an immunoglobulin located on the surface of B cells, is responsible for specific antigen recognition and binding (69). BCR regulates glucose utilisation by promoting glucose uptake and the expression of the GLUT1 transporter in B cells, leading to a rapid and sustained increase in glucose metabolism that provides essential energy and the foundation for growth. After BCR activation, there is a significant increase in glycolysis. Previous studies have demonstrated that enhanced glucose utilisation primarily involves the PI-3K signalling pathway associated with BCR activity. This pathway facilitates precise regulation of glucose utilisation within B lymphocytes through the PI-3K/AKT signalling cascade, ensuring their capacity to meet the energy demands essential for growth-related processes (70) (**Table 2**).

**Table 2:** Main metabolic patterns of inflammatory cells and their effects on disease.

Main metabolic patterns of inflammatory cells and their effects on disease				
cell type	Metabolic pattern	Effects in lesions		
		sample	Intervention method	result
M1 macrophages	glycolysis PPP	macrophages from mice and patients with atherosclerotic lesions within plaque	IFN-γ stimulation	M1 polarisation; Increased secretion of inflammatory cytokines, chemokines (18)
M2 macrophages	TCA FAO	anti-inflammatory alternatively activated macrophages	Etomoxir inhibits FAO; Oligomycin inhibits OXPHOS; FCCP inhibits uncoupled mitochondrial respiration	Mitochondrial oxidative metabolism is directly involved in M2 macrophage polarisation (11, 71, 72)
CD4^+^ T cell	glycolysis	CD4^+^ T cells and Tregs from Glut1 transgenic mice	Etomoxir stimulation	Th1, Th2, and Th17 cells primarily use glycolysis Tregs primarily use lipid metabolism (48)
Treg cell	FAO			
B2 cell	glycolysis	B cells from the mouse spleen	Incubated quiescent B cells with 1 mM 2-DOG along with anti-Ig	glycolytic flux is necessary for BCR-induced B-cell growth (70)

PPP, pentose phosphate pathway; IFN-γ, Human Interferon-γ; TCA, tricarboxylic acid cycle; FAO, fatty acid oxidation; OXPHOS, oxidative phosphorylation; Treg cell, regulatory T cell; BCR, B-cell receptor.

#### 2.1.2 Lipid metabolism: ApoA1 therapy suppresses Treg-to-T follicular cell conversion

During atherosclerosis, apolipoprotein A1 (ApoA1) indirectly affects T cell responses during inflammation. ApoA1 is the major protein component of high-density lipoprotein (HDL) and is produced in hepatocytes. Before being released into the plasma, it interacts with pre-HDL particles and ATP-binding cassette transporter A1 (ABCA1), acquiring phospholipids and cholesterol to form new HDL or ABCA1 (73). The formation of pre-HDL promotes the efflux of cholesterol from cells, resulting in a reduction in plaque volume. Research has shown that treating *ApoE* ^−/−^ mice with ApoA1 increased ABCA1 expression in Treg cells and restored cholesterol levels in these cells to normal (74). The anti-inflammatory properties of ApoA1 are also associated with changes in the lipid raft composition. Lipid rafts are microdomains on the cell membrane that are rich in sphingolipids and cholesterol, serving as enriched sites for IL-2 receptors. Lipid raft components regulate immune cell signalling and proliferation. Several studies have demonstrated that ApoA1 exerts regulatory effects on cholesterol levels, IL-2 receptor expression, and IL-6 expression in Treg cells during the progression of atherosclerosis, thereby impeding the transition from exTregs to Tfh cells and ultimately reducing atherosclerosis (73, 74).

Notably, diet-induced disruption of intracellular cholesterol metabolism is a significant factor affecting the differentiation and function of Tregs in atherosclerotic lesions. Maganto-García et al. (75) found that diet-induced hypercholesterolemia promoted the differentiation and migration of Treg cells in mouse splenic and prevented atherosclerosis. The classification of fatty acids into saturated, monounsaturated, and polyunsaturated forms is determined by the saturation level of their hydrocarbon chains. They can also be categorised as short-chain (SCFAs), medium-chain, or long-chain fatty acids (LCFAs) based on their carbon chain length. Fatty acids play a crucial role in regulating specific aspects of the body’s innate immunity and cholesterol metabolism within atherosclerotic plaques. The innate immune system relies on a diverse group of pattern-recognition receptors called toll-like receptors (TLRs) (76, 77). TLR4 is highly expressed in atherosclerosis and has multiple functions. It activates cell adhesion, enhancing the uptake of oxidized lipids by macrophages and foam cell formation (78, 79). It also influences cholesterol metabolism and its impact on atherosclerosis development (80). In contrast to saturated fatty acids, polyunsaturated acids do not activate the TLR4 signalling pathway (81–83) and instead inhibit NACHT-, leucine-rich repeat (LRR)-, and pyrin domain (PYD)-containing protein 3 (NLRP3) activity (84, 85). This is significant because NLRP3 may contribute to the pathogenesis of atherosclerosis through a signalling pathway that triggers PCSK9 secretion via IL-1β stimulation (86). LCFAs and SCFAs also play different roles in arteriosclerotic lesions. LCFAs can enhance the proliferation and differentiation of Teff cells into Th1 and Th17 cells, aggravating the progression of atherosclerosis (87). Conversely, dietary SCFAs affect Treg differentiation, which in turn improves and treats autoimmune-related diseases (87, 88).

These findings suggest the potential of using fat-free ApoA1 therapy and dietary adjustments to regulate the conversion between Treg and Tfh cells, offering potential benefits to patients with atherosclerosis and other inflammatory diseases.

#### 2.1.3 Amino acid metabolism: Alterations in glutamine, leucine, arginine, and tryptophan

Although glucose is generally considered the most important nutrient for inflammatory cells, amino acid metabolism also plays a crucial role in inflammatory cell proliferation and activation. Abnormal amino acid metabolism contributes to the occurrence and development of atherosclerotic lesions (89). Relevant studies mainly focus on monocytes and macrophages, with relatively fewer studies on lymphocytes.

Glutamine is one of the most widely studied amino acids involved in inflammation. It enters cell mitochondria through amino acid transporters and is converted to glutamic acid by the action of glutaminase. It also provides nutrients for the synthesis of other amino acids and NADPH and is an important energy source (90, 91). Glutamine, a non-essential amino acid in plasma, exerts intracellular effects on macrophage polarisation. Glutamine undergoes conversion to alpha-ketoglutaric acid (α-KG) through the enzymatic actions of glutaminase (GLS) and glutamate dehydrogenase (GDH/GLUD1). α-KG plays a crucial role in the tricarboxylic acid cycle, and its deficiency disrupts normal metabolic processes, thereby promoting the polarisation of macrophages towards the M1 phenotype and enhancing pro-inflammatory cytokine secretion. Conversely, active glutamine metabolism can stimulate macrophages to polarise towards the M2 type and secrete anti-inflammatory factors (9). Activated T cells exhibit increased glutamine uptake and metabolism, similar to cancer cells (90, 92). Depletion or deficiency of glutamine can disrupt T cell activation and proliferation (92). Rapid extracellular glutamine uptake depends on the amino acid transport weight group solute carrier family 1, member 5 (Slc1a5, also known as ASCT2). In mouse immune and autoimmune models, Slc1a5 defects impair Th1 and Th17 cell differentiation and thus affect inflammatory T cell responses (93). Under these circumstances, targeted therapy with glutamine hydrolase holds promise in preclinical models of cardio-cerebral vascular disease (94).

Leucine is an essential amino acid that affects the differentiation of Teff cells. Leucine entry into activated T cells requires the involvement of the L-leucine transporter (LAT1) (95). T cells lacking LAT1 cannot differentiate into Th1 or Th17 cells, even with appropriate polarising cytokines (96). Genetic defects in the leucine sensor sestrin 2 also limit T-cell activation and differentiation (97).

There is growing evidence that tryptophan metabolism is also involved in inflammatory responses. Overexpression of aminophenamide 2,3 dioxygenase 1 (IDO1) during inflammation can drive tryptophan consumption, produce bioactive metabolites, and control the interaction between general control non-derepressible 2 and specific receptors, thereby shifting cytokine production toward an anti-inflammatory phenotype. Conversely, the ablation of IDO1 promotes the pro-inflammatory effect of immune cells. The induction of IDO1 is associated with protection against atherosclerosis and increased plaque stability (98, 99). Other studies suggest that in addition to IDO1, other enzymes involved in tryptophan degradation, such as kynurenine 3-hydroxylase, are also involved in inflammation regulation, potentially increasing the instability of atherosclerotic plaques (100).

Arginine metabolism and its byproduct, nitric oxide (NO), play crucial roles in the early stages of atherosclerosis (101), such as involvement in immunity and affect the phenotypic polarisation of macrophages (102). Under inflammatory conditions, macrophages exhibit overexpression of inducible nitric oxide synthase (iNOS), thereby promoting arginine metabolism and subsequent NO production. Elevated NO levels can impede the repolarisation process from M1 to M2 by interfering with the electron transport chain (103). Additionally, arginine inhibits T-cell proliferation and impairs their ability to migrate to related chemokines, possibly contributing to its protective effect against atherosclerosis (104) (**Table 3**).

**Table 3:** Amino acid metabolism in inflammatory cells.

Amino acid metabolism	Cell type	Intervention method	Result
Glutamine	Macrophages were extracted from C57BL6/J mice	Cells were cultured in glutamine medium and inhibited by membranomycin (1 μM or 2 μM) with n-glycosylation	Promoted the polarisation of macrophages towards M2, and then secrete anti-inflammatory factors (9)
	Activated T cells	Cells were cultured in glutamine-deficient media	Decreased Th1 production of IFNγ and Th17 production of IL-17 (93)
Leucine	Activated T cells	Stimulated with the leucine antagonist NALA	Th17 differentiation was inhibited, but Th1 and Th2 polarisation were not affected (105, 106)
tryptophan	Arterial samples were obtained from 30 patients undergoing vascular surgery and the T cells in them were analysed	IDO-induced tryptophan degradation-dependent pathways	Inhibiting T cell activation and may prevent atherosclerosis (98)
Arginine	Macrophages	Arginine was increased *in vivo* models of mouse peritoneal inflammation and *in vitro* RAW 264.7 macrophages	Arginine is also involved in immunity and affects the phenotypic polarisation of macrophages (102)
	T cells	Female APOE-deficient mice were supplemented with high arginine (2mg/L) for 14 weeks	inhibiting T cell proliferation and impairs their ability to migrate to related chemokines (104)

### 2.2 Changes in the immune system affect EC metabolism

Vascular ECs play a crucial role in vascular homeostasis and disease. One of the earliest events of atherosclerosis development is the activation and dysfunction of ECs in vulnerable arterial regions. As atherosclerosis progresses, it exhibits characteristics such as the formation of a fibrous cap covering a lipid-rich necrotic core and the accumulation of leukocytes at the plaque’s periphery. These immune cells influence the phenotype of ECs and promote plaque instability. Although the endothelium was initially considered an inert and semi-permeable barrier between blood components and underlying endothelial tissues, it is now recognised as an organ with active metabolic functions that profoundly impact vascular homeostasis and atherosclerosis throughout life (107).

Metabolic pathways regulate angiogenesis, inflammation, and barrier function of ECs in atherosclerotic lesions. The following section outlines the changes in glycolysis and lipid metabolism observed in ECs within atherosclerotic plaques, as well as the regulation of EC homeostasis and function through Krüppel-like transcription factor 2 (KLF2) and yes-associated protein/the transcriptional coactivator with PDZ-binding motif (YAP/TAZ) signalling.

#### 2.2.1 Pfkfb3 and PKM2 induce angiogenesis by enhancing glycolysis in ECs

The formation of functional blood vessels and vascular plexuses, including processes like EC junction reorganisation, tip cell migration, stem cell proliferation, and phagocytosis (108–112), requires a substantial amount of energy in the form of ATP (108). Vascular sprouting relies on the differentiation of ECs into specialised subtypes, each with a specific function. Once the blood vessels are perfused, ECs transition into a quiescent state while being firmly anchored in the extracellular matrix. Blood vessels in which ECs germinate are regulated by genetic signalling and metabolic factors. ECs prefer to rely on glycolytic metabolism to minimise the production of ROS and rapidly produce ATP (95). Glycolytic enzymes and ATP are concentrated within the lamellar and filamentous pseudopods of ECs, facilitating their rapid motility (111).

Pfkfb3, the most prominent member of the Pfkfb family, plays a pivotal role in glycolysis by serving as the key enzyme (111). Conversely, the isozyme Pfkfb4 exhibits diminished kinase activity and can either stimulate or inhibit glycolysis (113). Signalling molecules known to induce germination in tip and stem cells, such as VEGF and FGF2 have been found to upregulate Pfkfb3 expression, subsequently promoting glycolysis. Furthermore, Pfkb3 also governs EC proliferation and influences their motility. *In vitro* and *in vivo* experiments have demonstrated that deactivating Pfkfb3 leads to reduced EC proliferation, impaired formation of filopodia/lamellipodia, and compromised directional migration, ultimately resulting in impaired vascular growth and branching in mice deficient in endothelial Pfkfb3 (114).

Alternatively, the Pfkfb3 blockade may reduce angiogenesis through other mechanisms. For example, in the context of tumour angiogenesis, lactic acid can activate HIF1α, upregulating vascular endothelial growth factor receptor 2 (VEGFR2) and promoting angiogenesis, or activate proangiogenic nuclear factors by inhibiting oxygen sensor PHD2 κB/IL-8 (115, 116). Reduced lactate levels after the Pfkfb3 blockade may inhibit angiogenesis through these mechanisms. Additionally, it has been proposed that Pfkfb3 may regulate cell proliferation through nuclear activity independent of glycolysis (117). However, further research is required to ascertain whether targeting Pfkfb3 can effectively reduce pathological angiogenesis.

Pyruvate kinases, which are involved in the production of pyruvate and ATP, are crucial for regulating glycolytic flux (109). PKM2, one of the PK isoenzymes located at the junctions of ECs expressing VE-cadherin, provides the material and energy required for promoting EC binding dynamics, migration, and proliferation through hyperactive glycolysis (118). Silencing PKM2 reduces ATP levels near EC junctions, affects the dynamics and internalisation of VE-cadherin at EC junctions, reduces the number of filopodia in endothelial tip cells, and ultimately disrupts EC junction remodelling, collective migration, and angiogenic germination.

#### 2.2.2 FAO promotes angiogenesis and EC homeostasis

ECs serve as gatekeepers for fatty acid transport. The fatty acids at atherosclerotic sites are associated with alterations in biophysical properties and membrane protein function within EC membranes. Polyunsaturated fatty acids not only serve as carbon sources for cultured ECs but are also regulated by ECs for transport to metabolically active tissues (119). Circulating fatty acids can be locally released from triglyceride-rich lipoproteins in the lumen of ECs via lipoprotein lipase-mediated lipolysis. Subsequently, they can enter ECs via passive diffusion or fatty acid transporters (120). The silencing or deletion of fatty acid-related genes can affect various EC functions, including migration capacity, vascular sprouting ability, and permeability regulation. Additionally, it can impact the activity of endothelial nitric oxide synthase (eNOS), leading to the production of excessive NO, an important vasodilator. Therefore, dysregulation of eNOS and excessive NO production can lead to pathological dysfunction and contribute to the progression of atherosclerosis (121).

#### 2.2.3 KLF2 and YAP-TAZ regulate EC homeostasis and function

Vascular ECs undergo different flow patterns depending on their location. For instance, the aortic arch, near the branching of the large ductal artery, and the tip of the coronary artery are exposed to oscillating shear stress known as disturbed flow (d-flow). In contrast, the larger curvature of the aorta and thoracic aorta experience high shear stress known as steady flow (s-flow). Exposure to d-flow increases the susceptibility of ECs to intima-media thickening and atherosclerosis (122). Among the numerous mechanosensitive transcription factors that differentially regulate vascular pathophysiology, our focus lies on KLF2 and YAP/TAZ.

##### 2.2.3.1 KLF2 affects EC function by regulating the expression of LOX-1 and HRD1

KLF2 expression was down-regulated in ECs exposed to d-flow. KLF2 is the primary activator of eNOS expression and other regulatory genes in the ECs, Its ability to maintain optimal expression and activity is indispensable for preventing pathological alterations in blood vessels, including thrombosis, oxidative stress, and inflammation (123). While early atherosclerosis is associated with Lectin-type oxidized low-density lipoprotein receptor 1 (LOX-1) expression. The imbalance between the excessive production of ROS and inadequate antioxidant defences in atherosclerosis leads to profound oxidative stress and the transformation of low-density lipoproteins (LDL) into highly atherogenic oxidized LDL (ox-LDL). These ox-LDL particles are subsequently deposited subcutaneously and bind to the clearance receptor LOX-1. This leads to an increased expression of cell adhesion molecules in ECs, promoting increased adhesion and migration of inflammatory cells into the intima. Concurrently, endothelial dysfunction worsens owing to increased vasoconstrictor production, increased ROS, and depletion of endothelial nitrogen oxide production (124). The activation of KLF2 is crucial for LOX-1 expression under shear stress. Downregulating KLF2 increases LOX-1 expression while overexpressing it inhibits LOX-1 upregulation. KLF2 regulates the degradation of 3-hydroxy-3-methylglutaryl reductase (HRD1), an E3 ubiquitin ligase, by binding to its promoter. HRD1 expression in atherosclerotic ECs is significantly reduced due to ox-LDL. Conversely, overexpression of HRD1 prevents ox-LDL-induced apoptosis in ECs (125).

**Figure 2:**
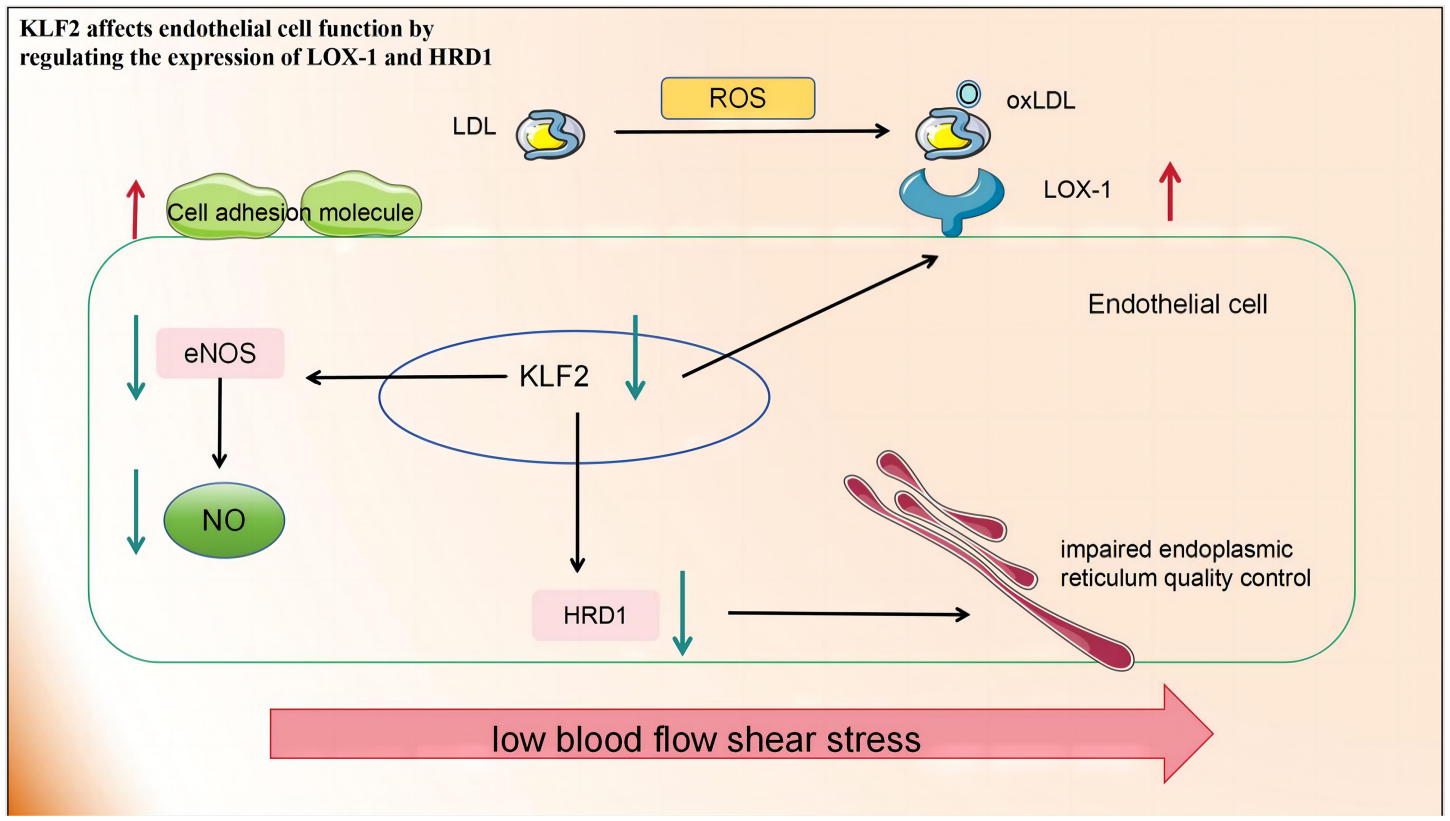
Low blood flow shear stress in atherosclerotic lesions leads to the downregulation of KLF2 in endothelial cells, thereby impacting endothelial cell function in the following aspects: 1) The production of eNOS and NO is diminished, consequently impairing their roles in promoting vascular relaxation and inhibiting inflammation. 2) The upregulation of LOX-1 expression on the cell membrane facilitates increased ox-LDL entry into cells, resulting in cellular damage. 3) The downregulation of HRD-1 expression involved in endoplasmic reticulum-related protein degradation pathway compromises endoplasmic reticulum quality control. KLF2, Recombinant Human Krueppel-like factor 2; eNOS, endothelial nitric oxide synthase; NO, nitric oxide; LOX-1, lectin-like oxidised LDL receptor 1; HRD-1, hydroxy-3-methylglutaryl reductase degradation.

In summary, KLF2 affects EC function by regulating the expression of various proteins and is a promising target for the treatment of atherosclerosis and related diseases (**Figure 2**).

##### 2.2.3.2 ​YAP/TAZ signalling modulates pathways that maintain EC quiescence and vascular homeostasis

On the contrary, YAP/TAZ was activated and translocated into the nuclei of ECs that were exposed to atherosclerotic interference in blood flow. Relevant findings were substantiated by Wang et al. through *in vivo* and *in vitro* experiments. they also found target genes such as angiogenesis inducer 61 (CYR61), connective tissue growth factor (CTGF), and ANKRD1 were upregulated. Mouse arterial surface analysis also revealed increased nuclear localisation of YAP/TAZ and elevated levels of endothelial target genes in atherosclerotic regions. In contrast, protective laminar flow inhibited YAP/TAZ activity. Knockdown of YAP/TAZ significantly reduced EC proliferation and the induction of pro-inflammatory phenotypes. Conversely, overexpression of YAP promoted EC proliferation and inflammation. Thus, inhibiting YAP/TAZ activation may be a promising therapeutic strategy for atherosclerotic protection. Notably, statins inhibit YAP/TAZ activity, thereby reducing disturbed flow-induced proliferation and inflammation (126).

**Figure 3:**
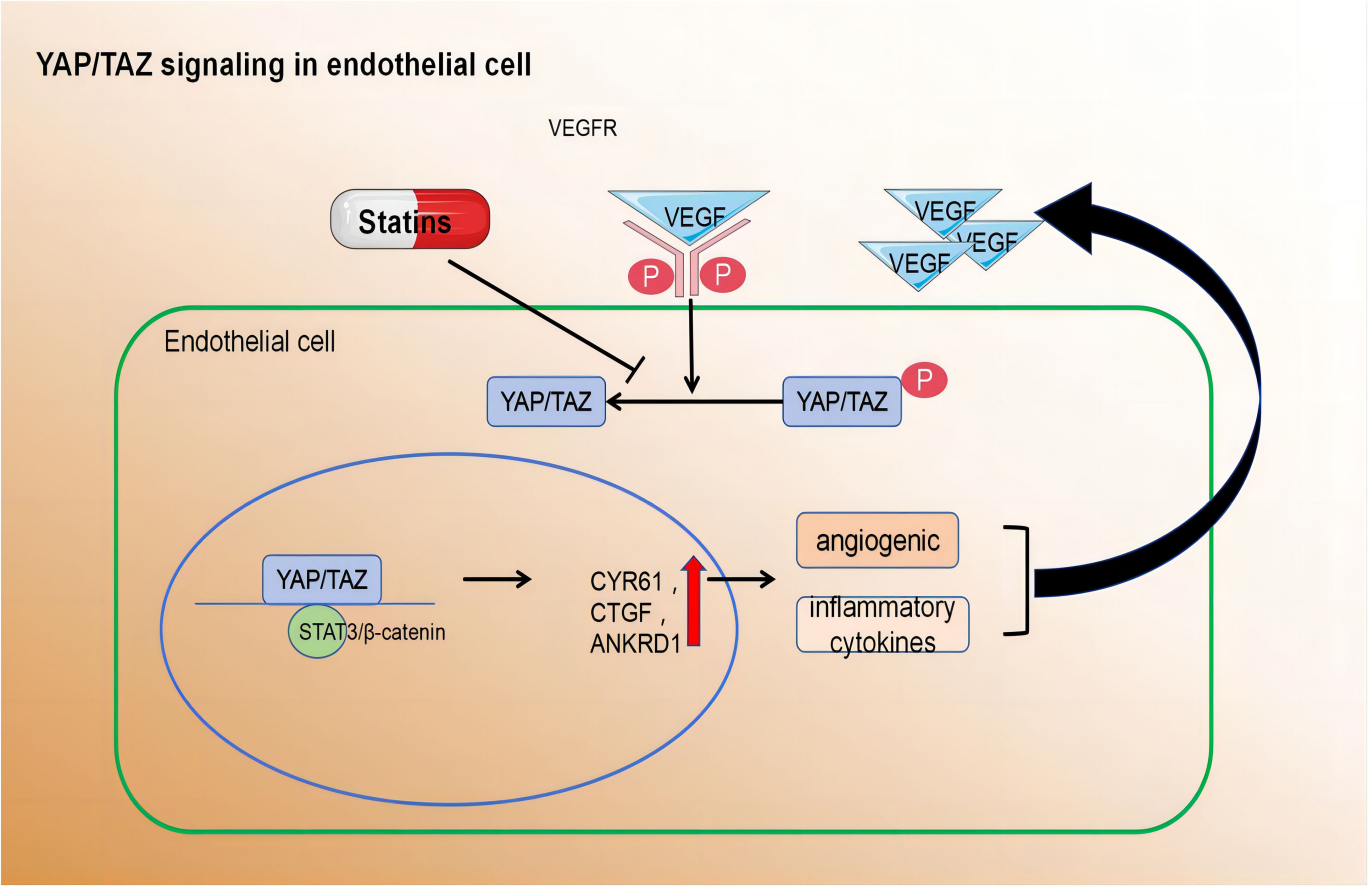
Pro-angiogenic growth factors, inflammatory cytokines, hypoxia, and disturbed blood flow activate YAP/TAZ leading to their translocation to the nucleus.Within the nucleus, YAP/TAZ interacts with STAT3 and β-catenin to induce transcription of downstream target genes. cysteine-rich angiogenic inducer 61 (CYR61), connective tissue growth factor (CTGF), and ankyrin repeat domain 1 (ANKRD1) Activated YAP/TAZ induce the expression of angiogenic and inflammatory cytokines.

In summary, the regulation of various EC functions is influenced by metabolic pathways, including those related to angiogenesis, inflammation, and barrier function. Despite advancements in understanding EC metabolism, many questions remain unanswered. A deeper comprehension of metabolic disturbances in ECs could lead to the development of novel therapeutic strategies for treating atherosclerosis (**Figure 3**).

### 2.3 Metabolic changes affect VSMCs function

The stability of atherosclerotic plaques depends on the thickness of the fibrous cap and the level of inflammation within it. Thinning of the vascular smooth muscle cell cap, which increases plaque rupture risk, is caused by cellular death and degradation of collagen and extracellular matrix (ECM). Vascular smooth muscle cells need to proliferate and synthesise new matrix components for effective repair. However, this process is hindered by cell death and senescence. The balance between cell proliferation, migration, death, and senescence plays a crucial role in determining the population size of vascular smooth muscle cells in atherosclerotic plaques. Understanding and regulating these processes are essential for maintaining stability during atherosclerosis development and plaque formation. Vascular smooth muscle cells are mainly found in the tunica media of arteries, exhibiting a mature “contractile” phenotype characterized by limited proliferation rates and expression of specific contractile proteins crucial for optimal vascular function (127). However, in response to vascular injury, these cells undergo a phenotypic transition from their static “contractile” state to a highly migratory and proliferative “synthetic” state. This shift significantly contributes to the development of Atherosclerosis, hypertension, and intimal hyperplasia formation (128–130). Recent research has revealed that metabolic reprogramming drives this transformation in VSMCs, involving key metabolic pathways such as glycolysis, fatty acid oxidation, and amino acid metabolism in both physiological and pathological vascular systems (130).

#### 2.3.1 Glycolysis and phenotypic changes in SMCs

During the development of atherosclerotic plaques, VSMCs transition from their contractile state to a more synthetic state, involving proliferation and migration from the tunica media to the intima. This phenotypic alteration is closely linked to alterations in glucose metabolism, particularly glycolysis (131). One key driver of VSMC proliferation in atherosclerosis is the upregulation of GLUT1, a glucose transporter. This upregulation results in a substantial increase (44%) in the intracellular glucose concentration within these cells (132–134). The higher glucose levels provide ample energy and lactic acid for further VSMC proliferation. Therefore, modulating glycolysis presents a promising therapeutic avenue for treating atherosclerosis.

Inhibition of the glycosylation pathway rate-limiting enzyme PKM2 leads to a decrease in extracellular and intracellular lactate production (135). Lactate dehydrogenase A (LDHA) converts pyruvate to lactic acid, which affects the survival, proliferation, migration, and invasion of human and rat aortic SMCs (133, 134). Additionally, LDHA down-regulates adenosine monophosphate-activated protein kinase (AMPK), which is involved in the vascular injury pathway. Furthermore, pyruvate can be oxidized to form acetyl-CoA by the PDH enzyme complex in the mitochondria of SMCs, entering the TCA cycle (136). Therefore, inhibition of PDK1 weakens the TCA cycle and shifts glucose metabolism from OXPHOS to glycolytic pathways (137).

In summary, the regulation of glycolysis is a key factor influencing VSMC proliferation. Additionally, VSMCs possess functional mitochondria, which not only produce energy but also provide metabolites necessary for biomass synthesis. Targeting mitochondrial complex I activity may reduce neointimal hyperplasia by inhibiting VSMC proliferation and migration (138). Furthermore, the presence of ox-LDL induces oxidative stress and ROS production in human VSMCs. High levels of ox-LDL exacerbate VSMC apoptosis, resulting in matrix and collagen loss and the thinning of the fibrous cap (139). These intricate metabolic processes play critical roles in the development of atherosclerotic plaques and offer potential therapeutic targets for intervention.

#### 2.3.2 Fatty acid metabolism affects SMC proliferation

In addition to glucose, VSMCs can also derive energy from fatty acids. FAO yields a higher amount of energy compared to glucose metabolism but requires higher oxygen levels. The presence of fatty acids can impact the utilisation of glucose and glycogen in both resting and contracting VSMCs (140). During the phenotypic transformation of VSMCs, there is a decrease in glucose oxidation and an increase in FAO. This elevation in FAO may provide VSMCs with additional energy for rapid proliferation, migration, synthesis, and secretion of the extracellular matrix (141, 142). The Randall cycle plays a pivotal role in altering the preference for utilising either glucose or fatty acids as fuel sources. This shift towards increased FAO impedes the oxidation of glucose (143). The Randall cycle has been implicated in regulating oxidative metabolism in muscle tissue, adipose tissue (143, 144), and brain energy balance (145). However, its influence on VSMC metabolism remains unclear. Exploring this aspect could present an intriguing avenue for future research.

Notably, dysfunctional FAO has been observed in plaques within the carotid artery of humans (146). This impaired FAO capacity may limit the functions of VSMCs, such as proliferation and migration, which are essential for plaque stability and vascular health.

#### 2.3.3 Amino acid metabolism regulates SMC function

Currently, there is a growing body of research focusing on the role of amino acid metabolism in VSMCs. Glutamine, the primary non-essential amino acid in plasma, plays a pivotal role in this process. Recent findings indicate that Slc1a5 plays a pivotal role in the efficient transportation of L-glutamine, and this transport mechanism has the potential to enhance VSMC proliferation (147). Moreover, glutamine is utilised to produce glutathione (γ-glutamyl-L-cysteinylglycine, GSH), an essential component involved in combating free radicals. Within VSMCs, both reduced GSH and its oxidized form (glutathione disulfide, GSSG) play critical roles in maintaining cellular redox balance (148). Depletion of GSH and increased DNA damage have been shown to inhibit growth and induce cell death in human VSMCs (149–151). Nitric oxide, a free radical in VSMCs, induces p53 expression and triggers programmed cell death by consuming intracellular GSH, making it a potent initiator of apoptosis (152). Additionally, it impedes mitochondrial respiration by suppressing the functions of complexes I and II; thereby affecting the relaxation of vascular smooth muscle (107). Furthermore, L-arginine effectively suppresses the proliferation and migration of VSMCs, even in the absence of NOS (153–155). Tryptophan, an essential amino acid serving as a substrate for serotonin synthesis, significantly enhances both proliferative and migratory tendencies of rat VSMCs under laboratory conditions (156). Elevated cysteine levels cause inflammation, oxidative stress, and increased proliferation and migration of VSMCs (157, 158). Notably, endogenous synthesis of hydrogen sulphide (H_2_S) from cysteine has protective effects on blood vessels. It inhibits NADPH oxidase, ROS production, glutathione disulfide formation, glutathione synthesis, and cysteine uptake (159, 160).

**Figure 4:**
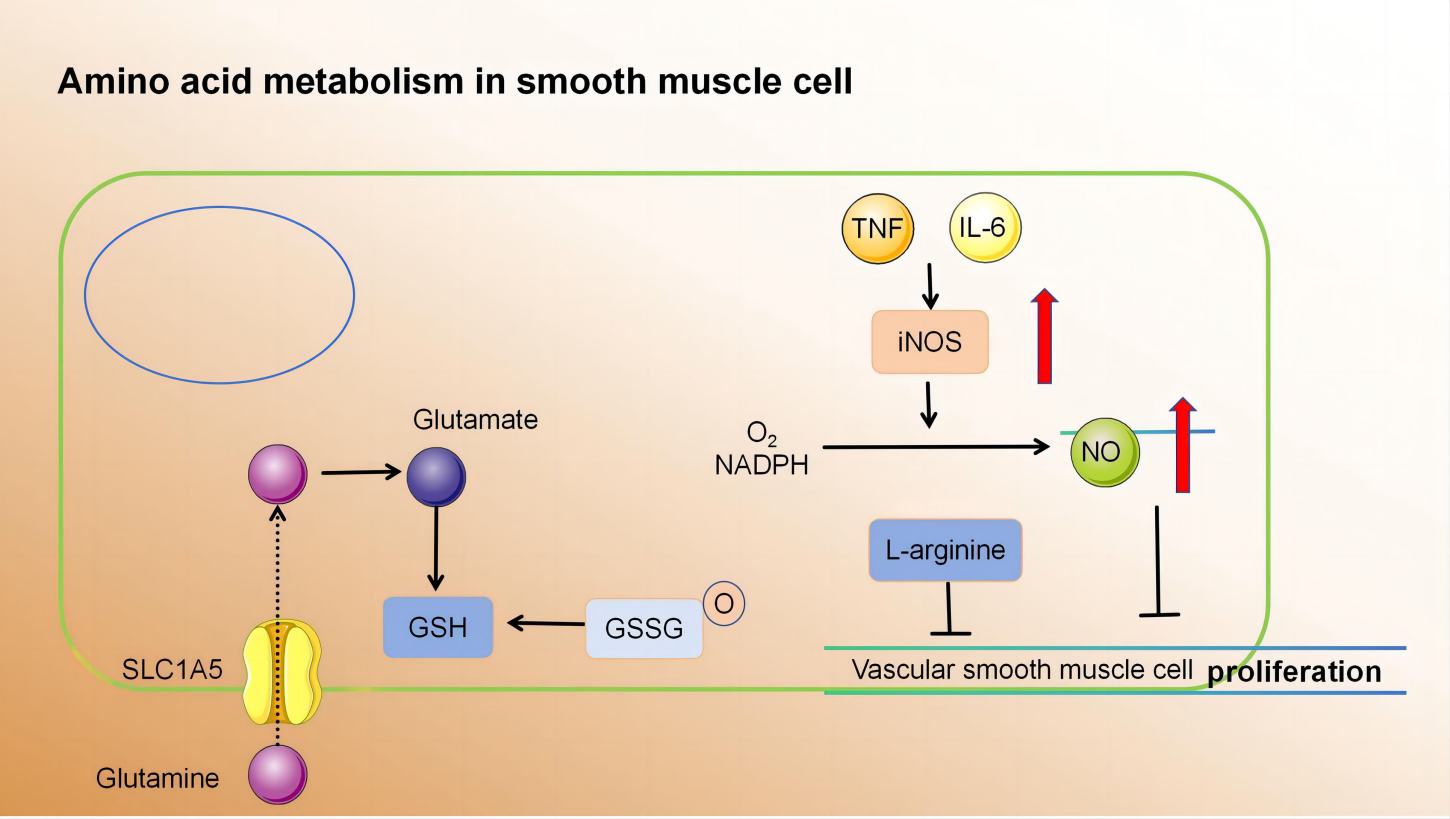
(1) Slc1a5 is a high-affinity L-glutamine transporter.glutamine absorption mediated by Slc1a5 can enhance the proliferation of VSMC. (2) In VSMCs, GSH and GSSG play crucial roles in cellular redox maintenance. (3) NO is a free radical. In VSMCs, NO activates programmed cell death through the consumption of intracellular GSH, thereby acting as a powerful apoptosis trigger. (4) L-arginine inhibits the proliferation and migration of VSMCs in the absence of NOS. Slc1a5, Solute Carrier Family 1, Member 5; VSMC, vascular smooth muscle cell; GSH, γ-glutamyl-L-cysteinylglycine; GSSG, Oxidized glutathione.

In summary, amino acid metabolism in atherosclerotic lesions affects smooth muscle proliferation and migration. These processes have profound implications for vascular health and the development of conditions such as atherosclerosis (**Figure 4**) (**Table 4**).

**Table 4:** Smooth muscle cell metabolism.

Metabolic pattern		Sample processing	Result or conclusion
Glycolysis		PDGF stimulates VSMCs in primary rat aorta	Promoting VSMC proliferation and migration (131)
Fatty acid metabolism		VSMCs were exposed to PDGF	Synthetic VSMCs demonstrated a 20% decrease in glucose oxidation, which was accompanied by an increase in fatty acid oxidation (141)
Amino acid metabolism	Glutamine	Slc1a5 is consumed by silencing RNA or blocking Slc1a5-mediated glutamine uptake	Inhibition of VSMC proliferation (147)
	Glutathione	Exogenous H_2_O_2_ depletion of GSH	VSMC growth inhibition and cell death were induced (149)
	L-arginine	L-arginine treatment of carotid artery injury rat model	L-arginine effectively suppresses the proliferation and migration of VSMC (153)
	Tryptophan	The 5-HT2BR antagonist acts on smooth muscle cells	Inhibition of VSMC migration (156)
	Elevated cysteine	High homocysteine stimulated carotid artery injury in rats	Promoting VSMC proliferation (157)

PDGF, platelet-derived growth factor-BB; 5-HT2BR, 5-HT receptor 2B; VSMC, vascular smooth muscle cell.

## 3 Effects of inflammatory reactions on arteriosclerosis plaques

### 3.1 Inflammatory cells interact with ECs during the initial stages of lesion development

In the initial stage of plaque development, there is an accumulation and aggregation of LDL and Very Low-Density Lipoprotein (VLDL) particles beneath the endothelium. These particles undergo oxidation and enzymatic modifications, resulting in the formation of oxidized phospholipids (oxPLS). The presence of oxPLS promotes inflammation and activates ECs. Simultaneously, various adhesion molecules are expressed, leading to the recruitment of white blood cells and platelets into the endothelial lining of blood vessels (161).

Monocytes firmly adhere to ECs through the interaction between monocyte integrins and ligands on ECs. Immunohistochemical analysis of human lesions and genetic studies in mice have demonstrated the significance of monocyte integrins VLA-4 and LFA-1, as well as their respective EC ligands VCAM-1 and ICAM-1, during the early stages of atherosclerosis. Moreover, platelet aggregation on the endothelium covering atherosclerotic lesions may enhance monocyte-EC interactions by inducing NF-κB signalling, promoting the expression of adhesion molecules, and depositing platelet-derived chemical factors on activated endothelium (162).

### 3.2 Inflammatory factors regulate the internal environment of atherosclerosis plaques

In addition to inflammatory cells, cytokines synthesised and expressed within atherosclerotic plaques play a significant role in shaping the internal environment for these plaques. Various stimuli trigger the release of inflammatory factors, including IL-1, IL-6, IL-8, IL-12, IL-18, soluble CD40 (SCD40), and TNF. These factors exert diverse effects that contribute to increased vascular permeability caused by inflammation (SCD40), and TNF. These factors exert diverse effects that contribute to increased vascular permeability caused by inflammation (163).

Clinical research on anti-thrombotic therapy involving canakinumab has investigated the role of IL-1 in the induction of atherosclerosis. Monoclonal antibodies targeting IL-1 effectively inhibit the formation and progression of atherosclerotic plaques (164). Studies like MIRACL have demonstrated an association between stroke risk and levels of high-sensitivity C-reactive protein (hs-CRP), serum amyloid A protein (SAA), and the inflammatory marker IL-6 (165). A phase II clinical trial demonstrated the potential of ziltivekimab, an all-human monoclonal antibody targeting IL-6 ligand, to significantly reduce multiple biomarkers associated with systemic inflammation and thrombosis (166). Furthermore, elevated levels of IL-8 within atherosclerotic plaques promote the recruitment and migration of monocytes toward vascular ECs, resulting in firm adhesion (167, 168). CD40 is predominantly expressed in the pro-inflammatory M1 phenotype of macrophages (169), and plasma CD40 levels are correlated with carotid artery severity. TNF-α stimulates interstitial cells to induce the expression of various adhesion molecules and triggers the secretion of inflammatory cytokines and chemical factors, thereby enhancing the recruitment of activated white blood cells to affected areas (170). TNF-α is a pleiotropic cytokine that acts through two primary receptors, TNF-1 and TNF-2. TNF-1 mediates pro-inflammatory signals, apoptosis, and degeneration, while activation of TNF-2 by TNF-α induces anti-inflammatory and cytoprotective responses, leading to cellular proliferation, differentiation, angiogenesis, and tissue repair (171). Within plaques, there are also anti-inflammatory cytokines such as IL-10 and TGF. TGF is a multifaceted late-stage cytokine with both protective and atherogenic properties. Vascular endothelial TGF generates positive signalling cascades that inhibit inflammation, reduce vascular permeability, and slow disease progression in hyperlipidaemic mice (172). The absence of TGF-β1 results in reduced VSMC differentiation within the body, accelerated lesion formation, and increased inflammation.

### 3.3 Characteristics of unstable plaques in advanced atherosclerosis

In advanced atherosclerotic plaques, the fibrous cap ruptures, exposing the necrotic core to thrombotic material, which initiates platelet aggregation and subsequent clot formation. The active release of plaque-derived cytokines, proteases, and coagulation/thrombosis-related factors further promotes the progression of vulnerable plaques (173).

In unstable atherosclerotic plaques, the thickness of the fibrous cap is reduced owing to decreased collagen synthesis in SMCs and/or enhanced collagen degradation in fibroblasts. This thinning promotes the development of vulnerable plaques. Reduced abundance of VSMCs in vulnerable plaques can contribute to decreased collagen synthesis. In areas of vulnerable plaque where apoptotic cells are present, macrophages can attenuate collagen production in VSMCs without inducing cell death by secreting lower levels of TGF-β, a key stimulator of collagen synthesis in SMCs. Additionally, macrophage-derived matrix metalloproteinases (MMPs), which refer to a group of enzymes responsible for activating proteins and breaking down different types of extracellular matrix proteins, can also contribute to weakening the fibrous cap (174).

Unstable plaques are characterised by the presence of a necrotic core, which results from programmed cell death of mature macrophages and impaired phagocytic ability to engulf dying macrophages in advanced plaques (175). Early atherosclerotic lesions efficiently clear apoptotic macrophages through phagocytosis, leading to minimal compromise in cellular integrity, and limited plaque progression. Apoptotic macrophages play a crucial protective role through three essential mechanisms (1): eliminating cells before they release harmful substances into the surrounding environment; (2) inducing an anti-inflammatory response mediated by IL-10 and TGF-β; (3) enhancing cell survival by counteracting internal toxic factors. The benefits of apoptotic macrophages encompass efficient cholesterol esterification and removal, elimination of proapoptotic oxidized lipids, and activation of AKT and NF-κB signalling pathways. However, in the advanced stages of atherosclerotic lesions characterised by oxidative stress and increased inflammation, macrophage endocytosis signalling pathways are impaired. This impairment leads to secondary necrotic cell death, and elevated levels of inflammation lead to cytotoxicity (176).

**Figure 5:**
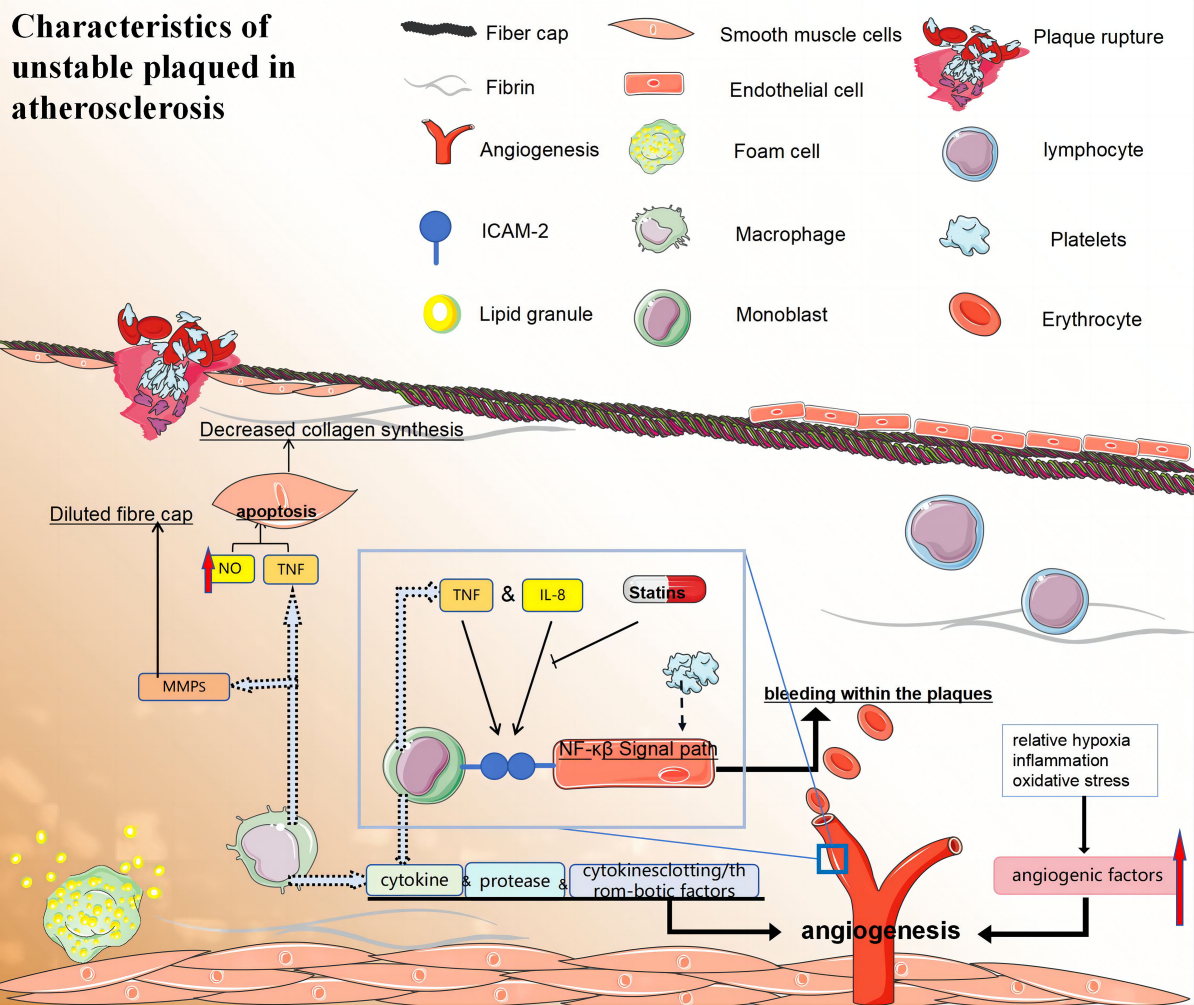
(1) Macrophages promote the formation of vulnerable plaques by secreting cytokines, proteases, and clotting/thrombotic factors. (2) i:The fibrous caps of late unstable atherosclerotic plaques became thinner. VSMCs may promote the formation of vulnerable plaques by reducing collagen synthesis and/or leading to collagen degradation. ii:Macrophages activate their apoptotic pathways and secrete TNF that is preapoptotic α, and that nitric oxide can trigger the apoptosis of SMC cells, which leading to a decrease in the number of VSMCs. iii:Macrophage-derived MMPS may also be involved in the dilution of fibre caps. (3) Specific local conditions (relative hypoxia, inflammation, and oxidative stress) in atherosclerotic plaques can also induce classical and non-classical angiogenic factors and promote neovascularisation. Neovascularisation increases bleeding within the plaques, which may lead to their instability and rupture. Monocytes firmly attach to endothelial cells through ICAM-1 interactions, while atherosclerotic lesions can enhance monocyte-endothelial interactions by activating NF-κB signaling. (4) Statins also exhibit anti-inflammatory properties by decreasing the expression of intercellular adhesion molecule-1 and inhibiting IL-6 secretion in monocytes and macrophages stimulated by LPS. (5) TNF:tumor necrosis factor; NF-κB:nuclear factor-κB; ICAM-1, intercellular cell adhesion molecule-1; MMPS, matrix metalloproteinase.

The low-oxygen, inflammatory, and oxidative stress environment in atherosclerotic plaques can induce both conventional and unconventional angiogenic factors, promoting the formation of neovascularisation (177). The presence of neovascularisation in the shoulder region is typically characterised by an incomplete and immature structure, rendering it prone to leakage, which contributes to intra-plaque haemorrhage (IPH). In 1936, it was postulated that repetitive IPH is implicated in the progression of atherosclerosis and thrombosis (178). Magnetic resonance imaging (MRI) studies on human carotid atherosclerosis over the past two decades have confirmed histological observations and indicated a significant influence of IPH on plaque evolution (179). Moreover, the potential of 18F-FDG as a functional imaging technique for identifying vulnerability by analysing signals associated with plaque histology has been suggested. However, concerns regarding the accuracy and precision of PET in detecting fragile plaques remain significant. Combining 18F-FDG-PET imaging with dynamically enhanced MRI is expected to enhance the diagnosis of plaque fragility in the future (**Figure 5**).

## 4 Mechanism of action, side effects, and drug combinations of statins

### 4.1 Statins stabilise plaques by acting on the inflammatory system

HMG-CoA reductase plays a crucial role in the production of cholesterol and was identified as a target enzyme for statins in the 1980s. Statins exert a wide range of effects beyond their primary function of lowering LDL cholesterol levels. Statin therapy has been associated with inflammatory responses triggered by stimulants such as oxidized LDL.

These drugs affect the function of various cells within atherosclerotic plaques through different signalling pathways. Statins enhance macrophage-mediated cholesterol esterification, increase the uptake and degradation of LDL, and promote NO synthesis in ECs. Additionally, they reduce inflammation, improve EC function, and inhibit SMC proliferation and apoptosis. These effects contribute to enhanced stability within atherosclerotic plaques, which, in turn, leads to the restoration of platelet activity and the clotting cascade. Therefore, statins are the most effective drugs for reducing lipid levels and mortality rates in patients with coronary issues. They significantly decrease the incidence of atherosclerosis in both primary and secondary prevention settings. Commonly prescribed statins include lovastatin, pravastatin, fluvastatin, simvastatin, pitavastatin, rosuvastatin, and atorvastatin.

### 4.2 Pharmacological mechanisms of statins

#### 4.2.1 Inhibition of HMG-CoA reductase

Statins exert their pharmacological effects through a multi-faceted approach. Primarily, they act on hepatocytes and inhibit the activity of HMG-CoA reductase, a critical enzyme responsible for the synthesis of mevalonate, a precursor to cholesterol. This leads to a decrease in cellular cholesterol levels. Moreover, statins promote the removal of sterol regulatory element-binding proteins (SREBPs) from the endoplasmic reticulum by inducing protease activity. SREBPs are responsible for increasing LDL receptor expression when they translocate to the cell nucleus. The decreased cholesterol levels in liver cells lead to an increase in the number of LDL receptors on their membranes, which facilitates the efficient clearance of LDL cholesterol particles from the bloodstream. Recent research suggests that statins may also enhance the expression of PCSK9, an enzyme involved in LDL receptor breakdown, potentially affecting their effectiveness in reducing LDL-C levels and preventing coronary heart disease risk (180).

#### 4.2.2 Statins reduce the sensitivity of LDL to oxidation and inhibit NLRP3 the activation of inflammasomes and the TLR signalling pathway

Several mechanisms underlie the antioxidant properties of statins (1): Statins reduce cholesterol levels, leading to decreased lipoprotein cholesterol and oxidative substrate levels (181). (2) They inhibit superoxide production in macrophages, thereby reducing cellular oxygen production. Additionally, statins inhibit the isopentenylation process of the p21 Rac protein in ECs, thereby impeding the formation of superoxide anions (182). This preserves the functionality of the endogenous antioxidant system, counteracting LDL oxidation (183). (3) By binding with phospholipids on lipoprotein surfaces, statins obstruct the penetration of free radicals generated during oxidative stress into the core region of lipoproteins. (4) Metabolites resulting from statin usage exhibit potent antioxidant capabilities that effectively protect against lipoprotein oxidation.

Ox-LDL promotes the activation of the NLRP3 inflammasome and its receptor pathway in plaques. The first signal is triggered by pattern recognition receptors such as TLR, which activates the NF-κB pathway, leading to the transcription of NLRP3 and other pro-inflammatory cytokines. The second signal involves the oligomerisation of activated NLRP3, ultimately forming the inflammasome, which activates pro-inflammatory cytokines. Statins exert a pleiotropic effect on the NLRP3 complex. They cause a reduction in TLR agonists and inhibit the TLR4/MyD88/NF-κB pathway. Additionally, statins inhibit the NLRP3 inflammatory response via the LOX-1/NF-κB pathway (184).

### 4.3 Effects of statins on intracellular cell functions in plaques

#### 4.3.1 Statins affect EC functions by regulating cholesterol esterification

The development of endothelial dysfunction due to elevated cholesterol levels is an early event in the progression of atherosclerosis. Hindered by hypercholesterolemia, the capacity of ECs to generate NO, a pivotal regulator of anti-atherosclerotic functions, is compromised. Research has demonstrated that statins effectively enhance endothelial function by reducing cholesterol levels. In 2002, Simionescu et al. (185) discovered that simvastatin alleviated the intracellular effects of LDL and restored endothelium-dependent relaxation, possibly due to increased NO synthesis. In 2018, Geng et al. (186) demonstrated that rosuvastatin protects endothelial cells in an *in vitro* model of human umbilical vein endothelial cells induced by ox-LDL, through its antioxidant function and up-regulation of the expression of eNOS, an endothelial protective factor. These findings confirm the advantageous impact of statins on promoting eNOS expression and preventing LDL-induced suppression of eNOS expression (187).

Furthermore, ox-LDL induces ECs to generate adhesion molecules and selective proteins, thereby facilitating immune cell infiltration into the intima. Statin therapy inhibits EC adhesion and permeability while reducing white blood cell migration, ultimately mitigating the inflammatory response within plaques (188).

#### 4.3.2 Effects on inflammatory cells

Cytokines released by macrophages and lymphocytes influence endothelial function and promote SMC proliferation, collagen degradation, and thrombosis. Statins act by inhibiting the expression and activity of these cytokines in the pathogenesis of atherosclerosis. In hypercholesterolemic rabbits, atorvastatin has been demonstrated to reduce the presence of macrophages, monocyte chemoattractant protein-1 (MCP-1), and nuclear factor NF-κB activation within the intima layer (189). Moreover, statins exhibit anti-inflammatory effects on monocytes and macrophages by downregulating intercellular adhesion molecule-1 expression induced by LPS and suppressing IL-6 secretion. The presence of crystalline cholesterol is widely acknowledged in the scientific community to play a significant role in arterial inflammation. This inflammatory response occurs due to the activation of NLRP3 (or cryopyrin) inflammasomes by cholesterol, subsequently triggering caspase-1 and leading to the release of IL-1 family cytokines (190). Additionally, studies have demonstrated that statins can effectively reduce hs-CRP levels and potentially mitigate adverse events in patients, even in the absence of evident hypercholesterolemia (191).

#### 4.3.3 Effects on the proliferation, migration, and apoptosis of arterial SMCs

In atherosclerotic lesions, the proliferation, migration, and invasion of SMCs into the subendothelial layer can induce intimal hyperplasia, while the secretion of collagen fibres by SMCs can influence the thickness of fibrous caps within plaques. In 2000, Bellosta et al. (192) conducted a study using both *in vitro* and *in vivo* models. The findings suggest that fluvastatin, simvastatin, lovastatin, and atorvastatin exhibit dose-dependent inhibition of SMC migration and proliferation. Chandrasekar et al. (193) demonstrated that the pro-atherosclerotic cytokine IL-18 stimulates SMC migration in an MMP9-dependent manner, and atorvastatin inhibits this process. Zhou et al. (194) established a diabetic mouse model by utilising *ApoE* ^-/-^ mice and administered atorvastatin treatment. Subsequently, they quantified SMCs and collagen composition, revealing that atorvastatin effectively reduced the number of SMCs while promoting collagen fibre synthesis. Moreover, it resulted in diminished atherosclerotic plaque area and enhanced arterial plaque stability through modulation of the RAGE pathway. However, Palomino-Morales et al. (195) demonstrated that statins effectively attenuated the activity of RhoA in SMCs, consequently leading to a reduction in collagen expression. The differences among these findings may be attributed to the different animal models used. By 2021, Jo et al. (196) conducted experiments to elucidate the mechanism underlying the inhibitory effect of statins on SMC apoptosis. Upon stimulation by ox-LDL in atherosclerotic lesions, platelet-derived growth factors induce the proliferation and migration of VSMCs. Prolonged stimulation ultimately leads to VSMC apoptosis. Statins exert their inhibitory effects by suppressing p38 activation through autophagy, thereby attenuating intracellular ROS levels and preventing apoptosis.

In summary, statins can promote the stability of atherosclerotic plaques by inhibiting the proliferation, migration, and apoptosis of SMCs and by affecting the collagen secreted by SMCs.

#### 4.3.4 Effects on platelet activation

Hyperlipidaemia and atherosclerotic plaque formation are associated with increased platelet activation and blood hypercoagulability. Elevated LDL levels promote an increase in thromboxane A2 production, which augments platelet responsiveness. Statin treatment leads to a reduction in collagen- and fibrinogen-induced platelet aggregation and thromboxane production. In a clinical trial, Barale et al. (197) assessed the impact of simvastatin treatment on platelet aggregation response and inflammatory cytokine expression in patients with hypercholesterolemia for a duration of 2 months. The findings demonstrated that in addition to ameliorating lipid distribution, simvastatin treatment also attenuated platelet aggregation rate and reduced circulating levels of pro-inflammatory factors, endothelial markers, and platelet markers. These results indicate that statins effectively reduce lipids, inhibit platelet activation, and improve inflammation levels and EC dysfunction within atherosclerotic plaques associated with primary hypercholesterolemia (**Table 5**).

**Table 5:** Effects of statins on intracellular cell functions in plaques.

	Experimental operation	Result or conclusion
Effects on ECs	A single injection of LDL (4mg/kg, 48 h) induced endothelial injury in rats Endothelial injury was also induced by incubation with LDL (300 mg/L) or ox-LDL (100 mg/L) in ECV304 cells	Simvastatin protects the vascular endothelium against the damages induced by LDL or ox-LDL in rats or cultured ECV304 cells (187)
	Thirty adult male hamsters were divided in three groups (1): hyperlipemic hamsters (HH) fed with 3% cholesterol and 15% butter, (2) hyperlipemic animals (HS) treated daily for 16 weeks by gavage with 0.3-mg/kg simvastatin and (3) normal hamsters. The blood and tissues were collected for biochemical assays and structural analysis.	Simvastatin reduces transcytosis of LDL and is able to restore the endothelial-dependent relaxation by an increase in NO synthesis (185)
	Human pulmonary artery EC was treated with simvastatin (5μM, 24 h)	Statin inhibits EC adhesion and permeability while reducing white blood cell migration, ultimately mitigating the inflammatory response within plaques (198)
Effects on inflammatory cells	Atherosclerotic lesion in rabbit was treated with Atorvastatin (5 mg/kg/d)	Atorvastatin reduces the presence of macrophages, MCP-1, and nuclear factor NF-κB activation within the intima layer (189)
	Normal human PBMCs and THP-1 cells were cultured with inhibitors of HMGR (simvastatin), geranylgeranyltransferase (GGTI-298), farnesyltransferase (FTI-277), and/or caspase-1 (Z-VAD(Ome)-FMK)	Statin activates pro-IL-1 processing and IL-1 release by human monocytes (190)
	1095 patients with non-cardiogenic ischemic stroke were assigned to the pravastatin (n=545) or control groups (n=550), and the endpoints were serum hs-CRP reduction and stroke recurrence	Pravastatin treatment may reduce hs-CRP, and higher hs-CRP levels increase the risk of vascular events (191)
Effects on the arterial SMCs	Studied the ability of statins to arterial myocyte migration and proliferation using *in vitro* and ex vivo models	Fluvastatin, simvastatin, lovastatin, and atorvastatin exhibit dose-dependent inhibition of SMC migration and proliferation (192)
	SMC was pretreated with atorvastatin prior to the determination of IL-18-induced migration	IL-18 stimulates SMC migration in an MMP9-dependent manner, and atorvastatin inhibits this process (193)
	Atorvastatin was used to treat *ApoE* ^-/-^ DM models	Atorvastatin reduces the number of SMCs while promoting collagen fiber synthesis (194)
	Lovastatin treated primary SMC	Statin affects the production of extracellular matrix in SMCs, especially for type I collagen (195)
	Sustained high concentrations of rosuvastatin (100 ng/ml) stimulated VSMCs	Rosuvastatin reduces intracellular ROS levels through autophagy, leading to its vascular protective activity (196)
Effects on platelet activation	In hypercholesterolemic patients allocated to diet (n=20) or a 2-month treatment with diet plus 40 mg simvastatin (n=25)	Simvastatin treatment reduced platelet activation and subclinical inflammation and improved endothelial dysfunction (197)

MCP-1, monocyte chemoattractant protein-1; LDL, low-density lipoprotein; ROS, reactive oxygen species.

### 4.4 Side effects of statin therapy

Statins demonstrate favourable drug tolerance but are commonly associated with adverse reactions such as hepatotoxicity and myopathy. Hanai et al. (199) have proposed a direct explanation for statin-related muscle toxicity, suggesting that these drugs can induce the expression of the Atrogin1 gene in skeletal muscles, leading to cytotoxic effects combined with interference in muscle differentiation processes like insulin induction. Moreover, some studies, including the final assessment of the JUPITER trial, have raised concerns about an elevated incidence of diabetes as a potential risk associated with the use of statins (200). Additionally, reports of potential toxic effects such as proteinuria and haematuria have also emerged (201).

Statins are powerful lipid-lowering drugs that can reduce the incidence and mortality of atherosclerosis, and they have been widely used to prevent primary and secondary cardiovascular diseases for more than 25 years. Interestingly, while statins treat atherosclerotic lesions by regulating lipid metabolism, they also regulate the function of various cells in the plaque and the secretion and expression of certain cytokines, thereby influencing the inflammatory response and plaque stability. However, since there may be other factors contributing to atherosclerotic lesions, such as hypertension and diabetes, and statins have some side effects including myopathy, the effect of statin monotherapy may be limited, and patient compliance may be low. Therefore, in clinical practice, statins are often used in combination with other lipid-lowering, antihypertensive, hypoglycaemic, and anti-inflammatory drugs.

### 4.5 A combination of statins and other drugs

The future of reducing atherosclerotic disease lies in combining statins with other medications. For example, inhibition of the regulatory protein PSCK9 effectively reduces plasma LDL levels, making it an ideal complement to statins (202). Experts propose that the combination of rosuvastatin and ezetimibe is safe and effective for treating hypercholesterolemia or hyperlipidaemia, regardless of diabetes or cardiovascular disease status. The fixed combination of 40 mg rosuvastatin/10 mg ezetimibe has been approved and evaluated (203). Statins can also be combined with fibrates, niacin, and omega-3 fatty acids to lower triglycerides in atherosclerosis development. However, extensive clinical studies are needed to assess the impact on cardiovascular outcomes and risk reduction for patients with hypertriglyceridemia. Hypertension is the primary risk factor for intravascular atherosclerosis, wherein the combination therapy of antihypertensive and statin treatment exhibits superior efficacy compared to monotherapy with antihypertensive agents alone in hypertensive patients without complications (204). The synergistic effect of lipid-lowering and inflammation-suppressing therapies is evident. Dicarboxylic acid, commonly prescribed as an agent for lowering blood sugar levels, not only regulates macrophage function in atherosclerosis but also suppresses inflammatory responses. By combining dimethylformic acid with statins, inflammation can effectively be inhibited while simultaneously reducing blood sugar levels and lipids, thereby enhancing the therapeutic potential for treating atherosclerosis (204, 205). Furthermore, ongoing clinical trials are investigating alternative anti-inflammatory agents targeting the CRP/IL-6/IL-1 axis such as low-dose methylidene and colchicine (206). When combined with aggressive LDL-C therapy, this approach may become the standard treatment for most patients with atherosclerosis. Aspirin and statins are well-established treatments for both atherosclerosis and coronary heart disease due to their cohesive properties and effective reduction of inflammation.

## 5 Conclusions

This review provides a comprehensive overview of the metabolic regulation in immune, endothelial, and smooth muscle cells and their potential contributions to the pathogenesis of atherosclerosis. Cells exposed to hypoxic conditions undergo distinct alterations in energy metabolism, including augmented glycolysis, impaired fatty acid synthesis, and abnormal amino acid metabolism. Inflammatory processes and lipid accumulation within atherosclerotic plaques are intricately linked to cellular proliferation, migration, senescence, and apoptosis. Different inflammatory responses within plaques affect plaque stability.

Statins and their combinations play an important role in the treatment of atherosclerotic lesions. In this review, the main pharmacological mechanisms of statins and their effects on the function of various cells in atherosclerotic plaques were briefly discussed. Considering the limitations and potential adverse reactions of statins in the treatment of atherosclerotic lesions, a comprehensive regimen is required, which combines statins with other lipid-lowering, antihypertensive, hypoglycaemic, antiplatelet aggregating, and anti-inflammatory drugs.

Although previous animal experiments, *in vitro* experiments, and clinical data analyses have provided extensive research on specific metabolic pathways, products, and the mechanisms and applications of lipid-lowering drugs, our understanding of drugs regulating inflammatory responses and influencing the pathological and physiological processes of atherosclerotic plaques remains limited. The continuous accumulation of clinical research data, utilisation of proteomics analysis, and the integration of advanced technologies will aid in the development of a more robust theoretical framework. This will enhance our understanding of angiogenesis, inflammatory changes, lipid stability, barrier function, atherosclerosis metabolic mechanisms, and treatment-related knowledge. These efforts may lead to the identification of new drug targets for treating atherosclerosis and related diseases, the development of more promising treatment strategies, and the reduction of unnecessary side effects.

## Author contributions

LMZ: Writing – original draft, Writing – review & editing. DM: Conceptualization, Supervision, Validation, Writing – review & editing. LW: Conceptualization, Writing – original draft, Writing – review & editing. XS: Writing – original draft. LF: Writing – original draft. LCZ: Writing – original draft. YC: Writing – original draft. YH: Writing – original draft. XW: Writing – original draft. JF: Writing – review & editing.

## Glossary

**Table:**

EC	endothelial cell
VSMC	vascular smooth muscle cell
LDL	low-density lipoprotein
OXPHOS	oxidative phosphorylation
ATP	Adenosine triphosphate
PPP	pentose phosphate pathway
FAO	fatty acid oxidation
NADPH	nicotinamide adenine dinucleotide phosphate
ROS	reactive oxygen species
PFKFB	phosphofructose-2 kinase B
PKM2	pyruvate kinase M2
TCA cycle	tricarboxylic acid cycle
Teff cell	effector T cell
Treg cell	regulatory T cell
TNF	tumor necrosis factor
IFN	Human Interferon
IL	Interleukin
TGF-b	transforming growth factor-b
PDK1	Pyruvic acid dehydrogenase kinase 1
LDH	lactate dehydrogenase
PDH	pyruvate dehydrogenase
HDL	high density lipoprotein
ABCA1	ATP binding cassette transport A1
HIF1a	hypoxia inducible factor 1
Slc1a5	Solute Carrier Family 1, Member 5
IDO1	indoleamine 2,3dioxygenase 1
iNOS	inducible nitric oxide synthase
Pfkfb3	phosphofructose-2-kinase/fructose 2,6-diphosphatase 3
eNOS	endothelial nitric oxide synthase
KLF2	Recombinant Human Krueppel-like factor 2
LOX-1	lectin-like oxidised LDL receptor 1
HRD1	hydroxy-3-methylglutaryl reductase degradation
NRF2	Nuclear Factor erythroid 2-Related Factor 2
HO-1	heme oxygenase-1
NF-kB	nuclear factor kB
d-flow	disturbed blood flow
s-flow	stable flow
ECM	extracellular matrix
LDHA	lactate dehydrogenase A
AMPK	Adenosine monophosphate-activated protein kinases
SAA	Serum Amyloid A protein
SR-BI	scavenger receptor class B type I
IPH	intra plaque hemorrhage
SREBPs	strip sterol regulatory element-binding proteins
GLUT1	glucose transporter 1
FOXP3	forkheadbox Protein 3
DCs	dendritic cells
BCR	B-cell receptor
ApoA1	Apolipoprotein A1
pre-HDL	pre-high density lipoprotein
NLRP3, NACHT-	leucine-rich repeat (LRR)-, and pyrin domain (PYD)- containing protein 3
NO	nitric oxide
CPT1A	Carnitine palmitoyltransferase- 1A
ECM	extracellular matrix
